# Paeoniflorin Ameliorates Skeletal Muscle Atrophy in Chronic Kidney Disease *via* AMPK/SIRT1/PGC-1α-Mediated Oxidative Stress and Mitochondrial Dysfunction

**DOI:** 10.3389/fphar.2022.859723

**Published:** 2022-03-08

**Authors:** Qiang Li, Jing Wu, Jiawen Huang, Rong Hu, Haiyan You, Lingyu Liu, Dongtao Wang, Lianbo Wei

**Affiliations:** ^1^ Department of Traditional Chinese Medicine, Shenzhen Hospital, Southern Medical University, Shenzhen, China; ^2^ School of Traditional Chinese Medicine, Southern Medical University, Guangzhou, China; ^3^ Department of Rheumatology and Clinical Immunology, Zhujiang Hospital, Southern Medical University, Guangzhou, China; ^4^ First Affiliated Hospital of Henan University of Chinese Medicine, Zhengzhou, China

**Keywords:** paeoniflorin, chronic kidney disease, skeletal muscle atrophy, oxidative stress, mitochondrial dysfunction, AMPK/SIRT1/PGC-1α

## Abstract

Skeletal muscle atrophy is a common and serious complication of chronic kidney disease (CKD). Oxidative stress and mitochondrial dysfunction are involved in the pathogenesis of muscle atrophy. The aim of this study was to explore the effects and mechanisms of paeoniflorin on CKD skeletal muscle atrophy. We demonstrated that paeoniflorin significantly improved renal function, calcium/phosphorus disorders, nutrition index and skeletal muscle atrophy in the 5/6 nephrectomized model rats. Paeoniflorin ameliorated the expression of proteins associated with muscle atrophy and muscle differentiation, including muscle atrophy F-box (MAFbx/atrogin-1), muscle RING finger 1 (MuRF1), MyoD and myogenin (MyoG). In addition, paeoniflorin modulated redox homeostasis by increasing antioxidant activity and suppressing excessive accumulation of reactive oxygen species (ROS). Paeoniflorin alleviated mitochondrial dysfunction by increasing the activities of electron transport chain complexes and mitochondrial membrane potential. Furthermore, paeoniflorin also regulates mitochondrial dynamics. Importantly, paeoniflorin upregulated the expression of silent information regulator 1 (SIRT1), peroxisome proliferator-activated receptor gamma coactivator-1α (PGC-1α), and phosphorylation of AMP-activated protein kinase (AMPK). Similar results were observed in C2C12 myoblasts treated with TNF-α and paeoniflorin. Notably, these beneficial effects of paeoniflorin on muscle atrophy were abolished by inhibiting AMPK and SIRT1 and knocking down PGC-1α. Taken together, this study showed for the first time that paeoniflorin has great therapeutic potential for CKD skeletal muscle atrophy through AMPK/SIRT1/PGC-1α-mediated oxidative stress and mitochondrial dysfunction.

## Introduction

CKD has increasingly become a medical problem globally (Kalantar-Zadeh et al., 2021). The number of CKD patients accounted for 9.1% of the world’s total population in 2017, which resulted in 1.2 million deaths each year (GBD Chronic Kidney Disease Collaboration, 2020) and greatly increased the economic burden. In CKD patients, persistent imbalances between protein degradation and synthesis result in skeletal muscle atrophy, which is closely related to the rate of morbidity and mortality in CKD patients (Zhang et al., 2018; Teixeira et al., 2019; Watanabe et al., 2019). The prevalence of muscular protein mass in CKD ranges from 4% to 63% depending on the different operational criteria (Lamarca et al., 2014; D'Alessandro et al., 2018; Hanatani et al., 2018; Slee et al., 2020). Currently, there is still a lack of specific and effective pharmacological options for CKD skeletal muscle atrophy.

Numerous researchers have reported that inflammation, oxidative stress and subsequent mitochondrial dysfunction are important processes in the maintenance of skeletal muscle function in CKD (Watanabe et al., 2019; Chalupsky et al., 2021). The excessive accumulation of ROS has been proven to be an important link that leads to oxidative stress and mitochondrial dysfunction in CKD skeletal muscle atrophy (Abrigo et al., 2018). Oxidative stress is involved in the pathogenesis of cancer, CKD, chronic heart failure and diabetes mellitus (Uddin et al., 2021), which contribute to the degradation of myofibrillar proteins. Recently, studies have shown that mitochondrial dynamics play an important role in modulating mitochondrial morphology and maintaining mitochondrial homeostasis (Srinivasan et al., 2017).

Mitochondrial dynamics are regulated by mitochondrial fusion and fission processes (Tilokani et al., 2018). Mitochondrial fission is a crucial process in maintaining the mitochondrial network and relies on the cytosolic GTPase dynamin-related protein 1 (DRP1). DRP1 is dynamically recruited to the outer mitochondrial membrane and drives membrane constriction (Kraus and Ryan, 2017). The phosphorylation of DRP1 at serine 616 (p-DRP1[Ser616]) promotes mitochondrial localization and stimulates mitochondrial fission. Conversely, p-DRP1 (Ser637) represses DRP1 enzyme activity and inhibits mitochondrial fission (Cereghetti et al., 2008; Calì and Szabadkai, 2018; Fonseca et al., 2019). In addition, DRP1 mediates mitochondrial fission-1 (FIS1), mitochondrial fission factor (MFF) and mitochondrial fission process protein 1 (MTFP1), which are integral outer and inner mitochondrial membrane proteins (Loson et al., 2013; Wai and Langer, 2016). In contrast, mitochondrial fusion requires inner and outer mitochondrial membrane (IMM and OMM) GTPases. Outer mitochondrial membrane fusion is mediated by mitofusin 1 and mitofusin 2 (MFN1 and MFN2), whereas inner membrane fusion is mediated by optic atrophy 1 (OPA1) (Meyer et al., 2017; Romanello and Sandri, 2021).

PGC-1α maintains the energy homeostasis of cells, tissues and even the whole body by regulating multiple signaling pathways. Several studies have revealed that PGC-1α expression is reduced in skeletal muscle atrophy induced by diseases, including cancer, diabetes and CKD (Correia et al., 2015; Su et al., 2017; Kato et al., 2021). PGC-1α is controlled by mitochondrial energy metabolism-related regulators, such as AMPK and SIRT1. AMPK and SIRT1 act as important factors of oxidative stress and mitochondrial function in skeletal muscle and kidney disease (Cantó and Auwerx, 2009; Iwabu et al., 2010; Bhargava and Schnellmann, 2017). Therefore, the regulation of the AMPK/SIRT1/PGC-1α signaling pathways could be regarded as a potential drug target to combat muscle atrophy.

Paeoniflorin (PF), a monoterpene glucoside, is the major bioactive monomer and was extracted from Paeonia lactiflora Pall (Li et al., 2021). Paeonia lactiflora Pall has been used to treat pain, anemia, inflammation and immune disorders for more than 1,000 years as a traditional Chinese herb. The pharmacological effects of PF include anti-inflammatory, immune regulation, antioxidant, antitumor and neuroprotective effects (Zhang and Wei, 2020; Wang et al., 2021). Several previous studies have shown that PF improves oxidative stress and mitochondrial dysfunction via activation of AMPK signaling pathways (Zhu et al., 2018; Huang et al., 2021). Nevertheless, the effects and mechanism of PF on CKD skeletal muscle atrophy have not been clearly elucidated. In this study, we hypothesized that PF alleviates CKD skeletal muscle atrophy via AMPK/SIRT1/PGC-1α-mediated inflammation, oxidative stress and mitochondrial dysfunction.

## Materials and Methods

### Chemicals and Reagents

Commercially available PF was purchased from Chengdu Herbpurify Co., Ltd. (CAS: 23180-57-6, molecular formula: C_23_H_28_O_11_, HPLC >98%, Chengdu, China). Recombinant murine tumor necrosis factor (TNF)-α was obtained from R&D Systems Inc. (410-MT, Minneapolis, USA).

### Animals

Male Sprague–Dawley (SD) rats were obtained from the Experimental Animal Centre of Southern Medical University, China, certification number: SCXK 2016-0041, weighing 200 ± 20 g. The rats were housed at a fixed temperature (22 ± 2)°C and humidity (50 ± 20%) under 12 h/12 h light/dark cycles. All rats were allowed free access to the same specific-pathogen free (SPF) standard chow and water. All experiments and procedures were performed in accordance with a protocol approved by the Institutional Animal Care and Use Committee of Southern Medical University.

### Animal Model and PF Treatments

After a 1-week acclimatization period, all rats were randomly assigned to the 5/6 nephrectomized group or the sham-operated control group. The nephrectomized group rats underwent a 5/6 nephrectomy surgical resection of the upper and lower thirds of the left kidney and were subjected to right nephrectomy after 1 week. The Sham group rats were treated as described previously (Wang et al., 2019; Hu et al., 2020; Liu et al., 2021). Rats were observed postprocedure for 1–2 h, and their wound healing, movements, mental states and body weights were monitored weekly. The following is the procedure for determining the appropriate dosage: the recommended clinical dose of Radix Paeoniae Alba (RPA) (white peony) is 15 g/d for an adult (60 kg). In accordance with the 2020 Chinese Pharmacopoeia, the content of PF should not be less than 16 mg in 1 g (1.6%) of RPA (Chinese Pharmacopoeia Commission, 2020). Hence, the dose of PF should be 240 mg/60 kg, namely, at least 4 mg/kg for an adult. A human equivalent dose of the lower limit dose of PF 4 × 6.2 (the conversion coefficient) = a rat dose of 25 mg/kg (Nair and Jacob, 2016). The higher dose was set as 2 times the lower dose. Seven weeks after the operation, the CKD model was judged to be successful when the level of Scr in the 5/6 nephrectomy operation group was significantly higher than that in the sham operation group. Then, the successfully underwent 5/6 nephrectomy surgical operation rats were randomly divided into 3 groups (*n* = 10): CKD model group (Model group), CKD + PF low-dose group (PFL, 25 mg/kg) and CKD + PF high-dose group (PFH, 50 mg/kg). The rats in the two PF-treated groups were intragastrically administered 25 mg/kg or 50 mg/kg PF dissolved in distilled water once daily for 7 weeks. One rat in the Model group died at the 13th week, but none had died in the other groups.

### Specimen Collection

One day before sacrifice, the 24-h urine protein of each rat was collected in a metabolic cage. Blood samples, tibialis anterior (TA), gastrocnemius (GA) muscles and kidney tissues were collected from the anesthetized rats at the end of the study.

### Biochemical

Twenty-four-hour urinary protein was measured by Nanjing Jiancheng Biological Engineering Co., Ltd. (C035-2-1, Nanjing, China). The absorbance of the samples was recorded on a spectrophotometer (UV-2600/2700, Shimadzu). Hemoglobin (Hb) was analyzed using an auto hematology analyzer (BC-2800Vet, Mindray) from rat whole blood. The levels of Scr, BUN, albumin (ALB), serum calcium and serum phosphorus were assayed using an automated biochemical analyzer (Chemray 800, Rayto).

### Enzyme-Linked Immunosorbent Assay

To determine the effect of PF on rat inflammatory cytokines, such as serum TNF-α, interleukin (IL)-1β, IL-6 and IL-10, ELISA kits (Thermo Fisher Scientific Inc., USA) were used. The optical density (OD) at 450 nm was finally measured via a microplate reader (Multiskan™ GO, Thermo Fisher Scientific).

### Hematoxylin and Eosin Staining

TA and kidney tissues were fixed in 4% paraformaldehyde for 1–2 days and embedded in paraffin after dehydration. Then, the samples were sliced into 3- to 5-μm-thick sections, stained with H&E and observed using an optical microscope (Eclipse Ti-S, Nikon). The muscle fiber mean cross-sectional area (CSA) was measured in a blinded fashion, and two investigators assessed the images using ImageJ software.

### Masson’s Trichrome Staining

Masson’s trichrome staining (G1006, Servicebio, China) was performed in kidney paraffin sections following the manufacturer’s instructions.

### Sirius Red Staining

The TA paraffin sections were stained using a Sirius Red solution set (G1018, Servicebio) according to the instructions. Quantification of fibrosis was performed by Sirius Red staining using ImageJ software.

### Immunofluorescence Staining

GA sections were collected and routinely embedded in OCT solution (4583, Sakura). Frozen muscle samples were sliced into 8–10 μm sections. Next, antigen retrieval and BSA sealing were performed. Subsequently, the sections were stained with primary antibodies against the following proteins: MAFbx (1:100, A6825), MuRF-1 (1:100, A3101), and MyoD (1:100, A0671), which were purchased from Abclonal Technology, Inc. (Wuhan, China); MyoG (1:100, DF8273) was purchased from Affinity Biosciences. OH. USA at 4°C overnight. The sections were incubated with fluorescent secondary antibody (1:300, GB21303, Servicebio) in the dark for 1 h and then counterstained with DAPI (G1012, Servicebio). Finally, the sections were photographed with a fluorescence microscope (Eclipse Ti-S, Nikon). The fluorescence intensities were measured by ImageJ software.

### Immunohistochemical Staining

Briefly, paraffin sections (4 μm) were stained with primary antibodies, including anti-phospho-AMPKα (Thr172) (1:200, AF3423), anti-AMPKα (1:100, AF6423), anti-SIRT1 (1:100, DF6033) and anti-PGC-1α (1:100, AF5395), at 4°C overnight, which were purchased from Affinity. Positive staining was visualized using a DAB color development kit (G1211, Servicebio). Finally, the dyed sections were photographed with an optical microscope, and the positive staining area was calculated by ImageJ software.

### Transmission Electron Microscopy

The detailed procedures were based on our previous studies (Wang et al., 2019; Hu et al., 2020). Briefly, fresh TA muscles were trimmed into 1 mm^3^ blocks and quickly fixed in fixative solution. Then, the ultrathin sections (70 nm) were sliced (EM UC7, Leica). Images were captured using a transmission electron microscope (HT7800, HITACHI). Mitochondrial morphology was quantified by the mitochondrial area (μm) and Feret diameter (μm) of each mitochondrion per field using ImageJ software.

### Skeletal Muscle Mitochondrial Isolation

For muscle mitochondria, the GA muscles were processed as we described (Wang et al., 2020) by using a mitochondria isolation kit (C3606, Beyotime, Shanghai, China).

### Measurement of Mitochondrial Electron Transport Chain Enzyme Activities

Mitochondrial electron transport chain (ETC) enzyme activities, including Complex I (reduced nicotinamide adenine dinucleotide coenzyme Q reductase, A089-1), Complex II (succinate-coenzyme Q reductase, A089-2), Complex III (ubiquinol cytochrome c oxidoreductase, A089-3) and Complex IV (cytochrome c oxidoreductase, A089-4), were purchased from Nanjing Jiancheng. The protocol was adapted from the manufacturer’s protocol, incorporating aspects of our previously published work (Wang et al., 2020).

### Cell Culture and *in Vitro* Model

Mouse C2C12 myoblasts (Yaffe and Saxel, 1977) were obtained from the Chinese Academy of Science Cell Bank. Myoblasts were cultured in high-glucose DMEM (Gibco, New York, United States) supplemented with 10% fetal bovine serum (FBS, Gibco) and 1% penicillin–streptomycin (Gibco) in a humidified incubator containing 5% CO_2_ at 37°C. Cell media was changed every 24–48 h. Cells between the sixth and 10th generations were used for the following *in vitro* experiments. After the cells adhered, they were continuously incubated in fresh medium treated with or without PF (25 and 50 μM) and TNF-α (40 ng/ml) for another 48 h.

### Small Interfering RNA and Inhibitor Intervention

PGC-1α-targeted siRNA (PGC-1α-siRNA) and nonspecific-control siRNA (NC-siRNA) were obtained from Kidan Bio Technology Co., Ltd. (Guangzhou, China). The sequences of PGC-1α-siRNA and NC-siRNA are listed in Table 1. C2C12 cells were transfected with PGC-1α-siRNA or NC-siRNA for 24 h using Lipofectamine 3000 (Invitrogen, USA) following the manufacturer’s protocol. The AMPK and SIRT1 inhibitors Compound C (HY-13418A, Dorsomorphin) and EX-527 (HY-15452, Selisistat) were purchased from Med Chem Express Co., Ltd. (Shanghai, China). Cells were treated with TNF-α and PF for 24 h and then incubated with or without Compound C and EX-527 for another 24 h.

**TABLE 1:** The primer sequences used for RT-qPCR.

Gene name		Sequence (5′→3′)
NC-siRNA	sense	UUU​GUA​CUA​CAC​AAA​AGU​ACU​G
	anti-sense	GUA​CUU​UUG​UGU​AGU​ACA​AAU​U
PGC-1α-siRNA-1	sense	UUU​CUG​GGU​GGA​UUG​AAG​UGG​UGU​ATT
	anti-sense	UAC​ACC​ACU​UCA​AUC​CAC​CCA​GAA​ATT
PGC-1α-siRNA-2	sense	GGU​GGA​UUG​AAG​UGG​UGU​AGA​TT
	anti-sense	UCU​ACA​CCA​CUU​CAA​UCC​ACC​TT
PGC-1α-siRNA-3	sense	UCC​AGU​AAG​CAC​ACG​UUU​AUU​TT
	anti-sense	AAU​AAA​CGU​GUG​CUU​ACU​GGA​TT
MAFbx	sense	CAG​CTT​CGT​GAG​CGA​CCT​C
	anti-sense	GGC​AGT​CGA​GAA​GTC​CAG​TC
MuRF-1	sense	GTG​TGA​GGT​GCC​TAC​TTG​CTC
	anti-sense	GCT​CAG​TCT​TCT​GTC​CTT​GGA
MyoD	sense	CGG​GAC​ATA​GAC​TTG​ACA​GGC
	anti-sense	TCG​AAA​CAC​GGG​TCA​TCA​TAG​A
MyoG	sense	GAG​ACA​TCC​CCC​TAT​TTC​TAC​CA
	anti-sense	GCT​CAG​TCC​GCT​CAT​AGC​C
PGC-1α	sense	TAT​GGA​GTG​ACA​TAG​AGT​GTG​CT
	anti-sense	CCA​CTT​CAA​TCC​ACC​CAG​AAA​G
GAPDH	sense	ACT​CCA​CTC​ACG​GCA​AAT​TCA
	anti-sense	CGC​TCC​TGG​AAG​ATG​GTG​AT

### Cell Viability Assays

Cell viability of TNF-α and PF in C2C12 myoblasts was detected using a Cell Counting Kit-8 (CCK-8, Dojindo, Japan) according to the provided instructions. C2C12 myoblasts were plated in 96-well microplates at a density of 3 × 10^3^ cells/well in growth medium. TNF-α is a well-established model for inducing myotube atrophy *in vitro*. Cells were treated with different concentrations of TNF-α and PF at 37°C for 24 and 48 h after cell attachment. Compound C and EX-527 were incubated for 24 h at 37°C. Next, 10 µL of the CCK-8 solution was added to each well and incubated at 37°C for 45 min. The OD at 450 nm was then measured.

### Evaluation of Oxidative Stress and ROS

The activities of catalase (CAT, A007-1), glutathione peroxidase (GSH-Px, A005-1), superoxide dismutase (SOD, A001-3), malondialdehyde (MDA, A003-1) and the total antioxidant capacity (T-AOC, A015-2) were calculated by Nanjing Jiancheng. Mitochondrial ROS and intracellular ROS levels were measured by a H2DCFDA) fluorescent probe (S0033S, Beyotime). After centrifuging the muscle mitochondria suspension, the resulting precipitates were added to a freshly prepared 10 μM DCFDA solution and incubated at 37°C for 30 min in the dark. C2C12 myoblasts were seeded in 96-well microplates at a density of 5 × 10^3^ cells/well and treated as described previously in *Cell Culture and in Vitro Model* and *Small Interfering RNA and Inhibitor Intervention*. The rate of ROS generation in each well was measured at an excitation wavelength (Ex) of 488 nm/emission wavelength (Em) of 525 nm using a fluorescence microplate reader (Gen5, BioTek). Six representative images of intracellular ROS in different groups were also photographed with an inverted fluorescence microscope (Lax X, Leica).

### Mitochondrial Membrane Potential (Δψm) Determination

Δψm was evaluated in isolated muscle mitochondria or myoblasts (5 × 10^3^ cells/well) according to the instructions of the mitochondrial membrane potential assay kit (JC-1, BB-4105-3, BestBio, China). Red fluorescence (Ex 525 nm/Em 590 nm) represents aggregates of JC-1, whereas green fluorescence (Ex 490 nm/Em 530 nm) represents monomers of JC-1. The Δψm in each group was calculated as the ratio of JC-1 aggregates/monomers by a fluorescence microplate reader (Vanani et al., 2020).

### Evaluation of Adenosine 5′-Triphosphate, Lactate Dehydrogenase and Succinate Dehydrogenase

The adenosine 5′-triphosphate (ATP), lactate dehydrogenase (LDH) and succinate dehydrogenase (SDH) contents in mucles were determined in accordance with the manufacturer’s procedures (A095-1-1, A020-2, A022-1-1, Nanjing Jiancheng) by a spectrophotometer and microplate reader.

### Quantitative Real-Time Polymerase Chain Reaction

C2C12 cells were treated with or without PF (25 and 50 μM) and TNF-α (40 ng/ml) for 48 h in 24-well plates (5 × 10^4^cells/well). The detailed total RNA extraction and reverse transcription steps were performed as described previously (Wu et al., 2019; Liu et al., 2021) following the manufacturer’s kit instructions (RR047A and RR420A, TaKaRa). RT–qPCR was run on a StepOnePlus™ (Applied Bioscience, Thermo Fisher Scientific) Real-Time PCR System. Relative mRNA expression was calculated using the 2^−∆∆Ct^ method (Livak and Schmittgen, 2001). The primer sequences synthesized by Sangon Biotech are listed in Table 1.

### Western Blotting

Proteins were extracted from muscle tissues and C2C12 cells with RIPA lysis buffer (KGP702, KeyGen Biotech) on ice. Protein samples were then loaded onto 10% SDS polyacrylamide gels, and samples were transferred to PVDF membranes at 250 mA for 120 min. After blocking, the membranes were probed using the following primary antibodies at 4°C overnight: anti-MAFbx (ab168372), anti-AMPKα (ab32047), anti-PGC-1α (ab54481), anti-DRP1 (ab184247), anti-MFN1 (ab221661) and anti-MFN2 (ab124773) were from Abcam (Cambridge, UK); anti-MuRF-1 (A3101) and anti-MyoD (A0671) were from ABclonal; anti-MyoG (DF8273), anti-p-DRP1 (Ser616) (AF8470), anti-p-DRP1 (Ser637) (DF2980), anti-FIS1 (DF12005) and anti-MFF (DF12006) were from Affinity; and anti-p-AMPKα (Thr172) (#2535) and anti-SIRT1 (#9475) were from Cell Signaling Technologies (Danvers, MA, United States). Anti-MTFP1 (PAB42161) and anti-OPA1 (PAB36180) were purchased from Bioswamp (Wuhan, China). Phospho-antibodies were diluted to 1:500, while others were diluted to 1:1,000. GAPDH (1:5,000, #2118, Cell Signaling Technology) was used as a reference standard. The next day, the PVDF membranes were incubated with an HRP-conjugated secondary antibody (1:10,000, RM3002, Rayantibody) for 1 h. Finally, the immunoblots were visualized with an enhanced chemiluminescence (ECL) HRP substrate Kit (SignalFire^™^ ECL Reagent, #6883, Cell Signaling Technology) and captured in a ChemDoc™ XRS + system (Bio–Rad, USA).

### Statistical Analysis

Statistical analysis was conducted in GraphPad Prism Software 8.0 (CA, US). Data are summarized as the means ± standard deviation (S.D.) and tested for normality, which was then tested for homogeneity of variance through one-way ANOVA. Comparisons between two groups were performed with Student’s t test. Comparisons among multiple groups were conducted with one-way ANOVA. Differences were considered to be statistically significant at *p* < 0.05.

## Results

### PF Improved Renal Function, Calcium/Phosphorus Disorders and Nutrition Index in CKD Model Rats

**FIGURE 1:**
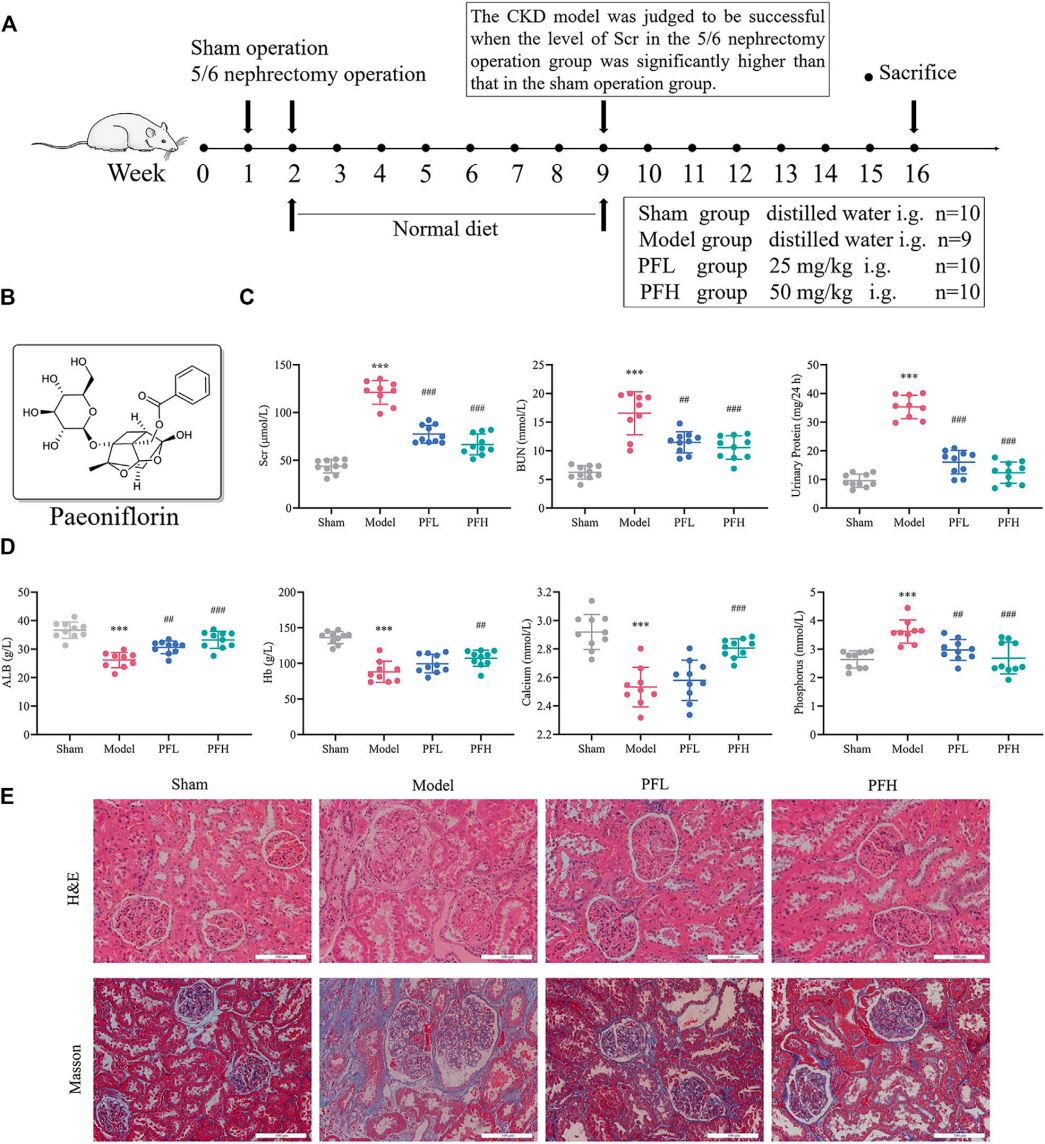
Paeoniflorin improved renal function, calcium/phosphorus disorders and nutrition index in CKD model rats. **(A)** Study design of the animal experiment and treatment. **(B)** Molecular structure of paeoniflorin (PF). **(C)** The levels of renal function indexes, including Scr, BUN and 24-h urinary protein. **(D)** The levels of serum calcium/phosphorus and nutrition indexes (Hb and ALB). **(E)** Representative images of kidney sections stained with H&E and Masson under the optical microscope (magnification: ×200, scale bars: 100 μm). The kidneys of CKD model rats showed glomerular sclerosis, focal renal fibrosis, enlargement of the tubular lumen, and infiltration of inflammatory cells. The data are expressed as the means ± standard deviation (S.D). Significant differences are indicated as ^*^ *p* < 0.05, ^**^ *p* < 0.01, ^***^ *p* < 0.001 vs. the Sham group. ^#^ *p* < 0.05, ^##^ *p* < 0.01, ^###^ *p* < 0.001 vs. the Model group. PFL, CKD model rats treated with a lower dose (25 mg/kg) of PF; PFH, CKD model rats treated with a higher dose (50 mg/kg) of PF.

The experimental timeline is presented in Figure 1A. The molecular structure of PF was drawn using ChemDraw 18.0 software (Figure 1B). The levels of Scr, BUN and 24-h urinary protein in the Model group were significantly higher than those in the Sham group. Conversely, the contents of ALB, Hb and calcium were significantly lower in the model rats than in the sham rats. Treatment with PF significantly improved these biochemical indexes, whereas the Hb and calcium levels were significantly higher in only the PFH group (Figures 1C,D). The concentration of serum phosphorus increased remarkably, and PF resulted in a reduced level of phosphorus (Figure 1D). H&E and Masson’s staining revealed a clear and complete kidney histological architecture in the Sham group. However, glomerular sclerosis, interstitial fibrosis and inflammatory cell infiltration were observed in the Model group. PF treatment reduced the occurrence of renal lesions and inhibited renal fibrosis caused by 5/6 nephrectomy surgery (Figure 1E).

### PF Inhibited Skeletal Muscle Atrophy in CKD Model Rats

**FIGURE 2:**
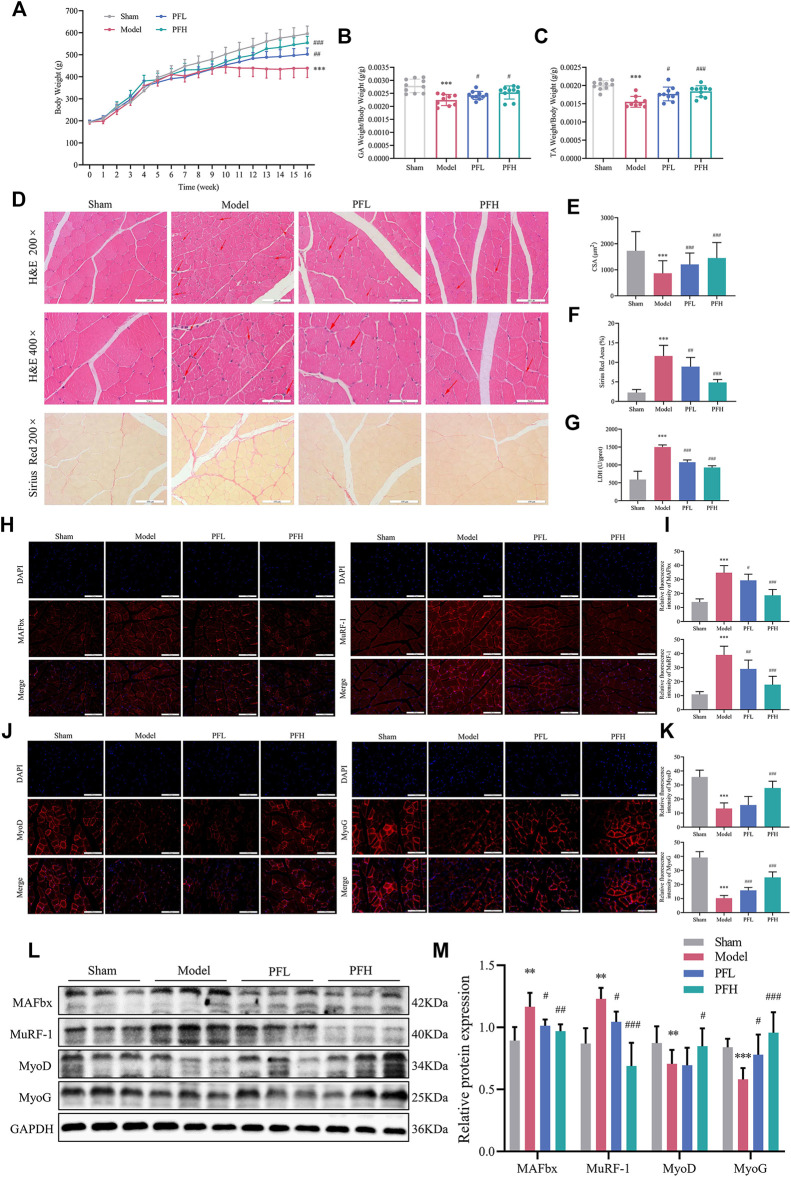
PF inhibited skeletal muscle atrophy in CKD model rats. **(A)** Body weight was evaluated weekly throughout the entire 16-weeks experimental period. **(B, C)** Weights of GA and TA muscles normalized by the final body weight. **(D)** Cross sections of TA muscle stained with H&E and Sirius Red. The red arrows indicate myofibers affected by atrophy, such as inflammatory cell infiltration, uneven muscle fiber thickness and cell nucleus displacement. Representative images of H&E staining (200× and 400×, scale bars: 100 and 50 μm). Representative images of Sirius Red staining (200×). **(E)** The cross-sectional area (CSA) of the TA muscle fibers of each group was measured (∼150 myofibers in each group). **(F)** The Sirius red area of the TA muscles (*n* = 15). **(G)** LDH levels in muscles. **(H)** MAFbx and MuRF-1 expression in the GA muscles was detected by immunofluorescence staining (200×, scale bars: 100 μm) using antibodies against MAFbx (red) and MuRF-1 (red), and the nuclei were detected via DAPI (blue) staining. **(I)** The relative fluorescence intensities of MAFbx and MuRF-1 were compared between the groups (*n* = 20). **(J)** Sections of GA muscle from different groups were examined with immunofluorescence staining (200×) using anti MyoD (red), anti MyoG (red) and DAPI (blue). **(K)** The relative fluorescence intensities of MyoD and MyoG were compared between groups (*n* = 20). **(L)** Representative immunoblotting of MAFbx, MuRF-1, MyoD, MyoG and GAPDH. **(M)** Quantification of protein expression in muscles (*n* = 6). The protein expression was normalized to that of GAPDH as a loading control. All of the data are expressed as the means ± S.Ds. Significant differences are indicated as ^*^ *p* < 0.05, ^**^ *p* < 0.01, ^***^ *p* < 0.001 vs. the Sham group. ^#^ *p* < 0.05, ^##^ *p* < 0.01, ^###^ *p* < 0.001 vs. the Model group.

Body weight, muscle weight and muscle fiber CSA were regarded as direct indicators of muscle atrophy. The PF-treated group showed obvious improvement in the final body weight (Figure 2A). GA and TA weights were quantified and normalized to the final body weights (Figures 2B,C). To study how PF affected the pathohistological features of muscle atrophy, H&E staining and Sirius Red staining of TA tissues were applied. The PF-treated group was confirmed by an increase in the fiber CSA and a decrease in the Sirius Red area of the muscles (Figures 2D–F). LDH is closely related to skeletal muscle injury. In the present study, PF significantly reduced muscle LDH levels (Figure 2G). The immunofluorescence staining and WB results showed that the Model group displayed an increase in the expression of muscle atrophy markers (MAFbx and MuRF-1), while the expression of myogenic differentiation markers (MyoD and MyoG) was attenuated. However, the exacerbation of muscle atrophy and suppression of muscle differentiation could be reversed by PF treatment (Figures 2H–M). These data indicated that PF efficiently inhibited CKD muscle atrophy.

### PF Alleviated Inflammation and Oxidative Stress in CKD Model Rats

**FIGURE 3:**
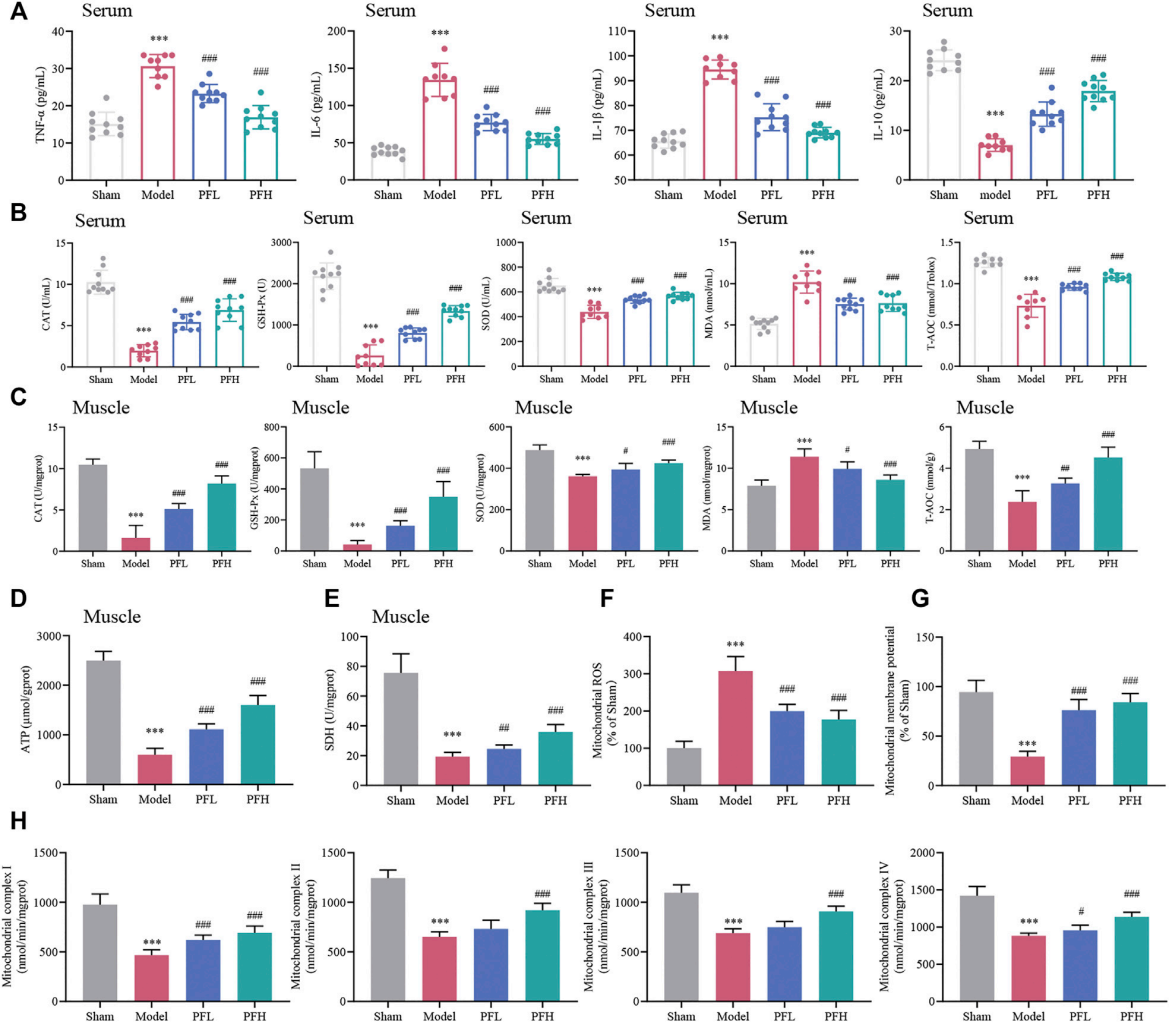
Effect of PF on inflammation, oxidative stress and mitochondrial dysfunction in CKD rats. **(A)** Expression of the inflammatory cytokines TNF-α, IL-6, IL-1β and IL-10 in serum. **(B, C)** CAT, GSH-Px, SOD, MDA and T-AOC contents in serum and muscles of different groups are presented in the histograms. **(D, E)** The expression of ATP and SDH in muscles. **(F)** PF inhibited mitochondrial reactive oxygen species (ROS) generation in muscles. **(G)** The effects of PF on mitochondrial membrane potential (Δψm) in muscles. **(H)** The activities of mitochondrial electron transport chain complexes I, II, III and IV in muscles between groups. All of the data are expressed as the means ± S.Ds. *n* = 6. Significant differences are indicated as ^*^ *p* < 0.05, ^**^ *p* < 0.01, ^***^ *p* < 0.001 vs. the Sham group. ^#^ *p* < 0.05, ^##^ *p* < 0.01, ^###^ *p* < 0.001 vs. the Model group.

PF exerted a dose-dependent effect on downregulating proinflammatory factors (TNF-α, IL-6, and IL-1β) and upregulating the anti-inflammatory factor IL-10 (Figure 3A), indicating that PF may improve the inflammatory response. CAT, GSH-Px, SOD, MDA and T-AOC are classic indicators for measuring the capacity to resist oxidative stress. The activities of T-AOC and antioxidant enzymes (CAT, GSH-Px and SOD) in the serum and muscles of rats in the Model group were decreased but partially increased after PF treatment. Correspondingly, the elevated MDA content in the Model group in both serum and muscles followed a downward trend after administration of PF (Figures 3B,C).

### Assay of ATP, SDH, Mitochondrial ROS, Δψm Levels and ETC Enzyme Activities in Skeletal Muscles of CKD Rats

The ATP, SDH and Δψm contents were decreased, and mitochondrial ROS generation was higher in the Model group than in the Sham group. However, these disadvantageous effects were ameliorated by PF treatment (Figures 3D–G). Additionally, the reduction in enzymatic activities of the mitochondrial ETC complexes (complexes I, II, III and IV) could be prevented by PF treatment (Figure 3H).

### PF Ameliorated Muscle Mitochondrial Structural Damage and Regulated Mitochondrial Dynamics in CKD Rats

**FIGURE 4:**
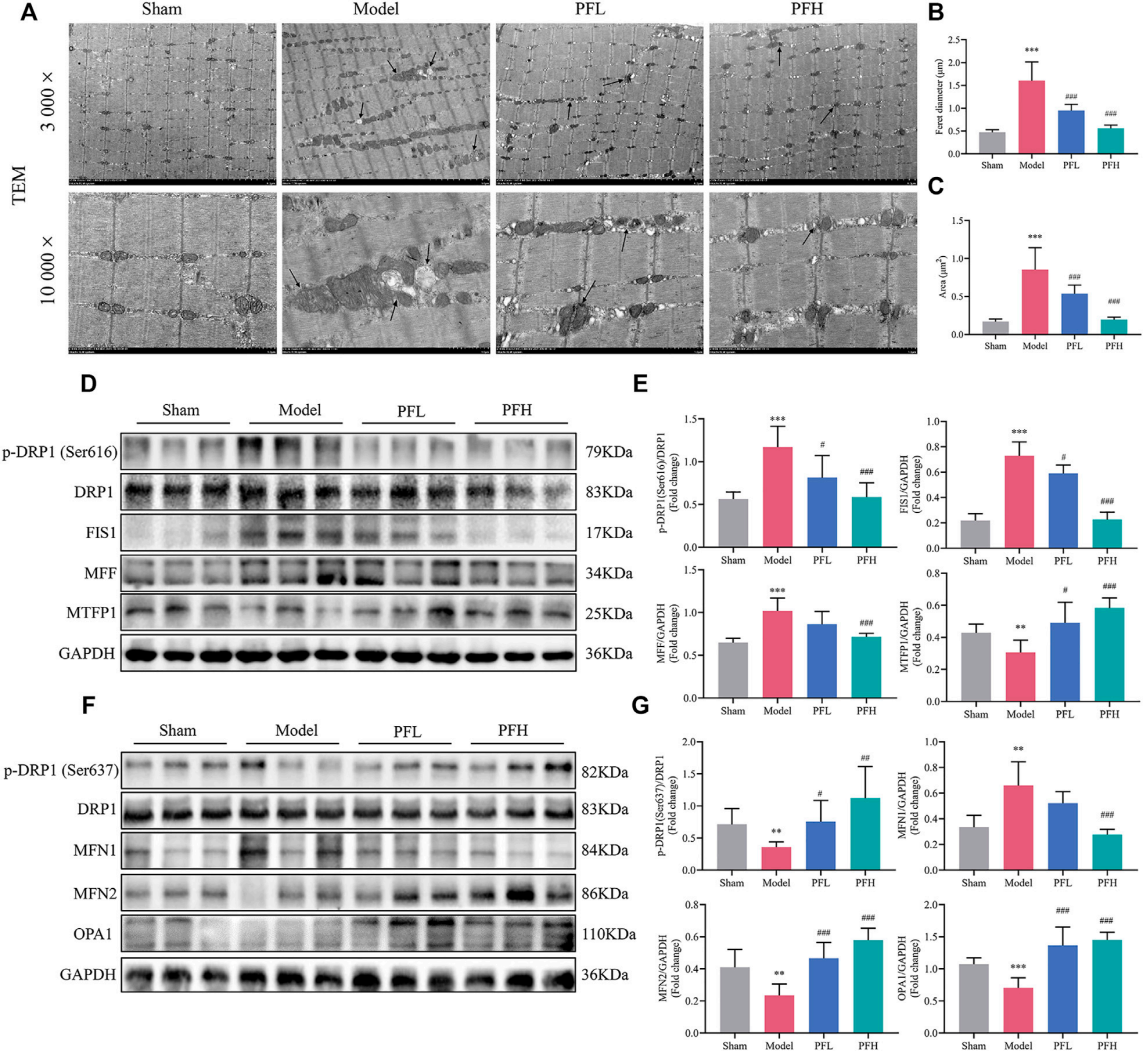
PF ameliorated mitochondrial structural damage and regulated mitochondrial dynamics in CKD rats. **(A)** Representative electron micrographs. Mitochondria were swollen and showed a disordered arrangement and membrane ruptures or large vacuoles in CKD model rats. The black arrows indicate mitochondria (magnification: 3,000× and 10,000×, scale bars: 5 and 1 μm). **(B, C)** The mitochondrial average Feret diameter and area from different groups in muscles were calculated (*n* = 20/group). **(D)** Representative western blots using antibodies against DRP1 phosphorylation at Ser616, DRP1, FIS1, MFF, MTFP1, and GAPDH. **(E)** Western blot quantification of the expression of mitochondrial fission-related proteins, including p-DRP1 (Ser616), DRP1, FIS1, MFF and MTFP1. **(F)** Representative western blots using antibodies against p-DRP1 (Ser637), DRP1, MFN1, MFN2, OPA1, and GAPDH. **(G)** The expression of mitochondrial fusion-related proteins. All protein expression was normalized to that of GAPDH as a loading control (*n* = 6). All of the data are expressed as the means ± S.Ds. Significant differences are indicated as ^*^ *p* < 0.05, ^**^ *p* < 0.01, ^***^ *p* < 0.001 vs. the Sham group. ^#^ *p* < 0.05, ^##^ *p* < 0.01, ^###^ *p* < 0.001 vs. the Model group.

Transmission Electron Microscopy (TEM) observations from skeletal muscles showed mitochondrial damage. The deleterious alterations in the morphology of mitochondria in the CKD model group mainly included indexes such as vacuolization, fractured or dissolved cristae, membrane loss, and swelling in skeletal muscles, which could be largely reduced by PF treatment (Figure 4A). Furthermore, the mitochondrial mean Feret diameter (μm) and enlarged area (μm^2^) were calculated (Figures 4B,C). In the mitochondrial dynamic system, the total DRP1 protein content remained unchanged in all groups. Nevertheless, the p-DRP1 (Ser616), FIS1 and MFF, which stimulate mitochondrial fission, was increased in the Model group, and these changes appeared to be attenuated with PF treatment. Interestingly, the expression of MTFP1 was downregulated, and these changes were impeded by PF treatment (Figures 4D,E). In contrast, p-DRP1 (Ser637), OPA1 and MFN2, which stimulate mitochondrial fusion, were decreased in the Model group, and these downregulations were hindered by PF administration. Interestingly, the level of MFN1 was significantly higher in the Model group, and these inblance of fisson–fusion were counteracted by PF treatment (Figures 4F,G).

### PF Enhanced the Expression of p-AMPKα, SIRT1 and PGC-1α in the Muscles of CKD Model Rats

**FIGURE 5:**
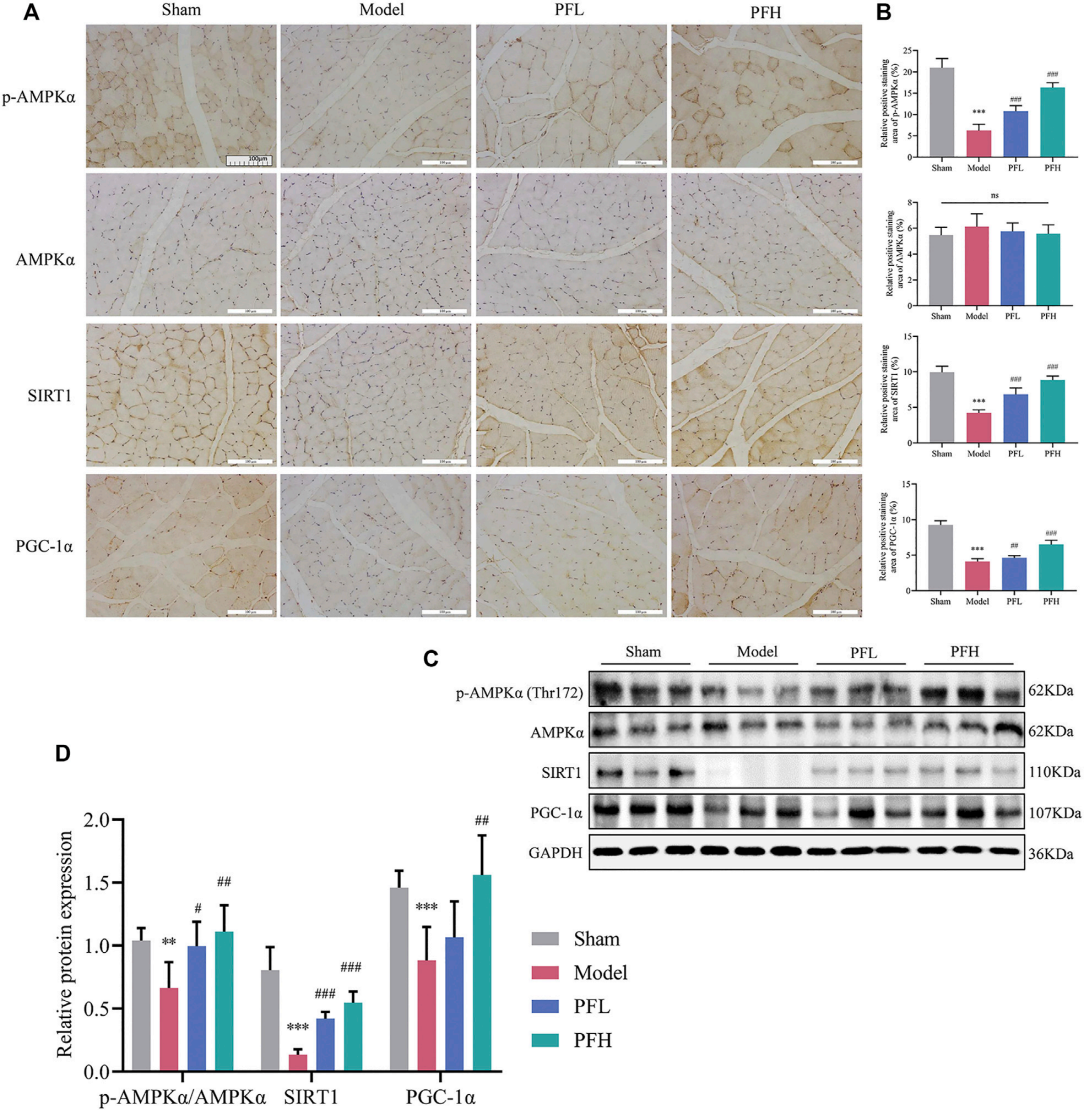
PF enhanced the expression of p-AMPKα, SIRT1, and PGC-1α in the muscles of CKD model rats. **(A)** Representative images of p-AMPKα, AMPKα, SIRT1, and PGC-1α expression in the TA muscles of rats using immunohistochemical (IHC) staining (magnification: ×200 scale bars: 100 µm). **(B)** The positive staining areas of p-AMPKα, AMPKα, SIRT1 and PGC-1α were compared between all groups (*n* = 10). **(C)** Representative immunoblots using antibodies against p-AMPKα (Thr172), AMPKα, SIRT1, PGC-1α and GAPDH. **(D)** Protein expression of p-AMPKα/AMPKα, SIRT1 and PGC-1α. The protein expression was normalized to that of GAPDH as a loading control (*n* = 6). All of the data are expressed as the means ± S.Ds. Significant differences are indicated as ^*^ *p* < 0.05, ^**^ *p* < 0.01, ^***^ *p* < 0.001 vs. the Sham group. ^#^ *p* < 0.05, ^##^ *p* < 0.01, ^###^ *p* < 0.001 vs. the Model group.

We evaluated whether PF could activate p-AMPKα (Thr172), AMPKα, SIRT1 and PGC-1α using IHC staining and immunoblotting. There was no difference in the total AMPKα protein content among the groups. However, p-AMPKα (Thr172), SIRT1 and PGC-1α levels were restored by PF in a dose-dependent manner (Figures 5A–D).

### PF Suppressed TNF-α-Induced C2C12 Myoblast Damage

**FIGURE 6:**
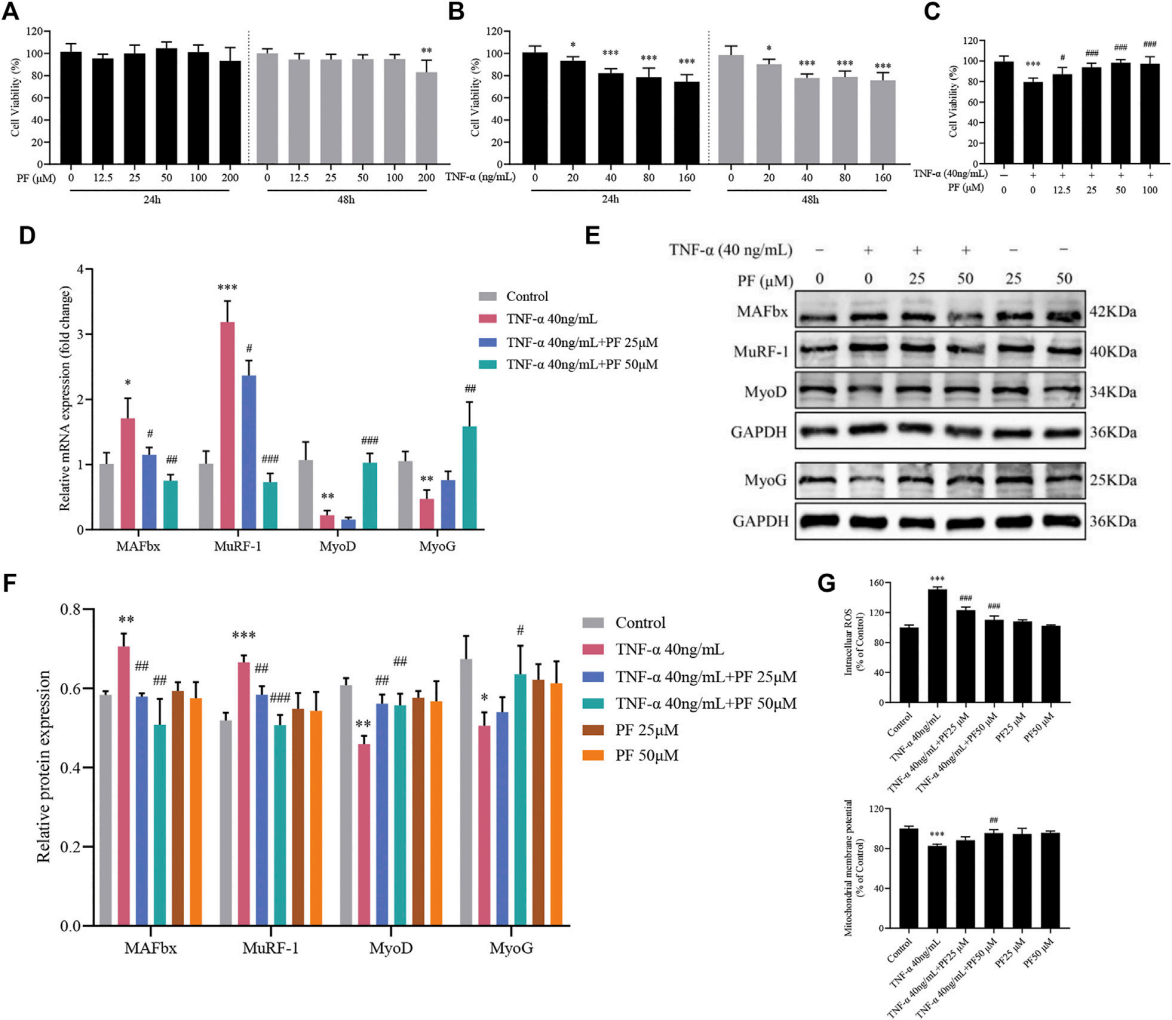
PF suppressed TNF-α-induced C2C12 myoblast damage. **(A)** C2C12 myoblasts were treated with TNF-α (0, 20, 40, 80 and 160 ng/ml) for 24 and 48 h. **(B)** Cytotoxicity of PF (0, 12.5, 25, 50, 100 and 200 μM) for 24 and 48 h. **(C)** Cells were treated with 40 ng/ml TNF-α and 12.5, 25, 50, and 100 μM PF for 48 h. Cell viability was measured with a CCK-8 assay (*n* = 6). ^*^ *p* < 0.05, ^**^ *p* < 0.01, ^***^ *p* < 0.001 compared with PF 0 μM or TNF-α 0 ng/ml. ^#^ *p* < 0.05, ^##^ *p* < 0.01, ^###^ *p* < 0.001 compared with the 40 ng/ml TNF-α group. **(D)** The expression of MAFbx, MuRF-1, MyoD and myoG was determined by RT–qPCR. **(E)** Representative western blots using antibodies against MAFbx, MuRF-1, MyoD, MyoG and GAPDH. **(F)** Quantification of protein expression. **(G)** PF inhibited intracellular ROS generation and increased Δψm in C2C12 cells. All protein expression was normalized to that of GAPDH as a loading control. The data are presented as the means ± S.D. *n* = 3, ^*^ *p* < 0.05, ^**^ *p* < 0.01, ^***^ *p* < 0.001 compared with the Control group. ^#^ *p* < 0.05, ^##^ *p* < 0.01, ^###^ *p* < 0.001 compared with the TNF-α 40 ng/ml group.

We next sought to extend our findings to evaluate the PF effects in C2C12 myoblasts. Figures 6A–C shows that incubation with TNF-α (40 ng/ml) and PF (25 and 50 μM) for 48 h displayed the best condition for further experiments in the C2C12 model. We found that PF greatly inhibited the expression of MAFbx and MuRF-1 compared with the TNF-α group, while the expression of MyoD and MyoG was enhanced at the mRNA and protein levels (Figures 6D–F). Furthermore, TNF-α induced intracellular ROS generation and Δψm reduction, which were reversed by PF treatment (Figure 6G). These results indicated that PF efficiently alleviated TNF-α-induced C2C12 cell damage, which was consistent with the *in vivo* results.

### PF Activated the AMPK/SIRT1/PGC-1α Signaling Pathway in C2C12 Myoblasts Induced by TNF-α

**FIGURE 7:**
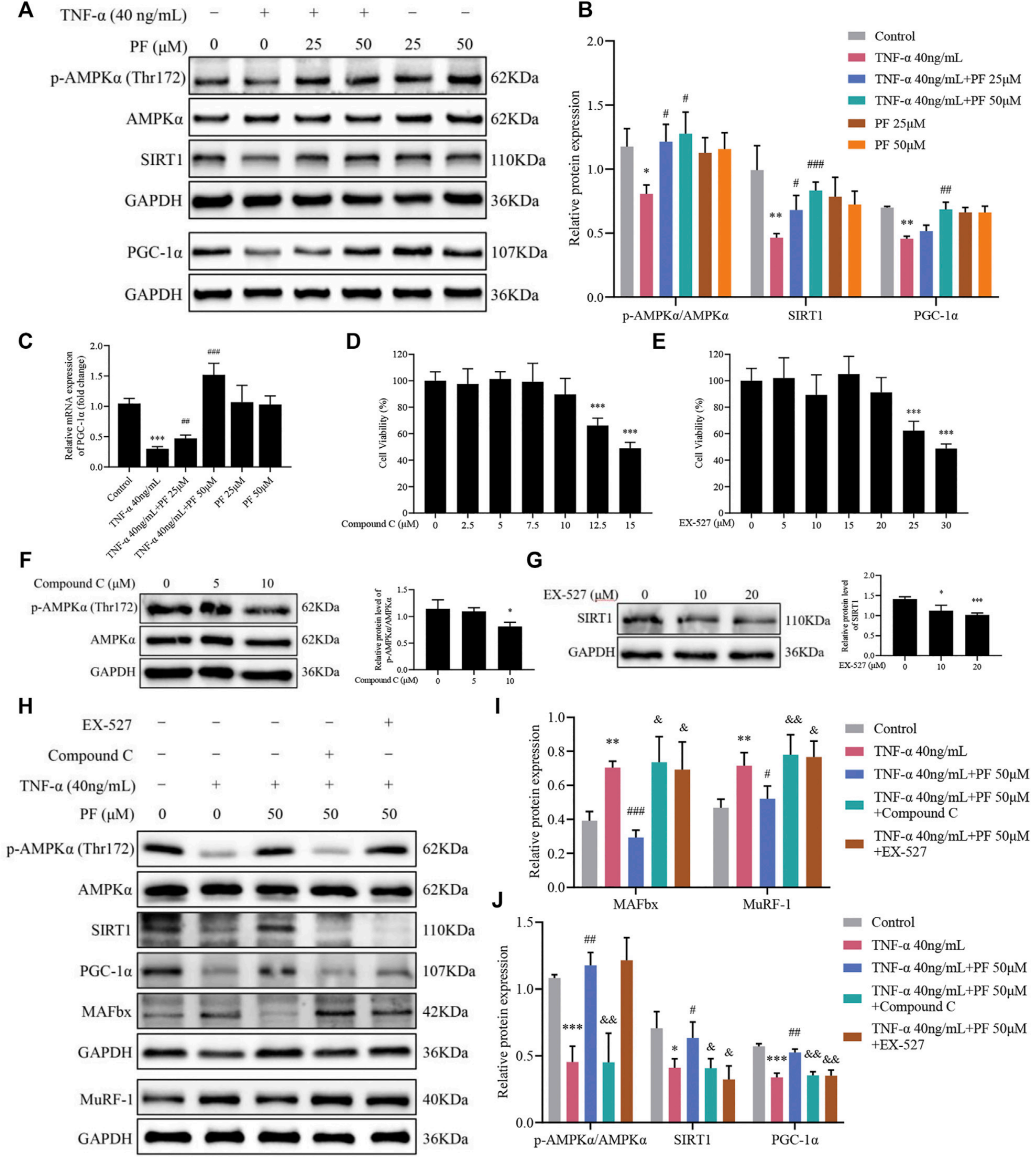
PF activated the AMPK/SIRT1/PGC-1α signaling pathway in C2C12 myoblasts induced by TNF-α. **(A)** Representative immunoblots using antibodies against p-AMPKα (Thr172), AMPKα, SIRT1, PGC-1α and GAPDH. **(B)** Quantification of protein expression. **(C)** The mRNA level of PGC-1α was assessed with RT–qPCR. **(D)** Cytotoxicity of Compound C (0, 2.5, 5, 7.5, 10, 12.5 and 15 μM) for 24 h (*n* = 6). **(E)** Cytotoxicity of EX-527 (0, 5, 10, 15, 20, 25 and 30 μM) for 24 h (*n* = 6). **(F)** The effect of different concentrations of Compound C on p-AMPKα/AMPKα protein levels. **(G)** The effect of different concentrations of EX-527 on SIRT1 protein inhibition. ^*^ *p* < 0.05, ^**^ *p* < 0.01, ^***^ *p* < 0.001 compared with the compound or EX-527 0 μM group. **(H)** Representative immunoblots using antibodies against MAFbx, MuRF-1, p-AMPKα (Thr172), AMPKα, SIRT1, PGC-1α and GAPDH. **(I, J)** Quantification of protein expression. All protein expression was normalized to that of GAPDH as a loading control. The data are presented as the means ± S.D. *n* = 3, ^*^ *p* < 0.05, ^**^ *p* < 0.01, ^***^ *p* < 0.001 compared with the Control group. ^#^ *p* < 0.05, ^##^ *p* < 0.01, ^###^ *p* < 0.001 compared with the TNF-α 40 ng/ml group, ^&^ *p* < 0.05, ^&&^ *p* < 0.01, ^&&&^ *p* < 0.001 compared with the TNF-α 40 ng/ml + PF 50 μM group.

Based on IHC staining and immunoblotting analysis in the CKD model, we further explored whether the effects of PF were related to the activation of the AMPK/SIRT1/PGC-1α signaling pathway in C2C12 myoblasts. Compared with controls, TNF-α treatment greatly hampered the expression of p-AMPKα/AMPKα, SIRT1 and PGC-1α, and PF treatment restored the levels of the three proteins (Figures 7A,B). Similarly, the mRNA results of PGC-1 showed the same trend (Figure 7C). To strengthen the opinion that PF inhibits muscle atrophy via AMPK/SIRT1/PGC-1α-mediated oxidative stress and mitochondrial damage, AMPK and SIRT1 inhibitors were used. Appropriate concentrations of AMPK and SIRT1 inhibitors were identified, 10 µM Compound C and 20 µM EX-527, respectively, for use in the following studies (Figures 7D–G). We observed that the expression of p-AMPKα/AMPKα, SIRT1 and PGC-1α was downregulated in the TNF-α+ PF+ Compound C group. Additionally, the protein expression of p-AMPKα was not obviously changed in the TNF-α+ PF+ EX-527 group. Meanwhile, we observed that the protective effects of PF against TNF-α-induced muscle atrophy were abolished in the TNF-α+ PF+ Compound C and EX-527 groups (Figures 7H–J). These data explain that PGC-1α is the downstream effector protein modulated by SIRT1 and AMPK. Knockdown of AMPKα or SIRT1 abrogated the protective effects of PF.

### PGC-1α Might Be Crucial in Mediating the Effects of PF on C2C12 Myoblasts Induced by TNF-α

**FIGURE 8:**
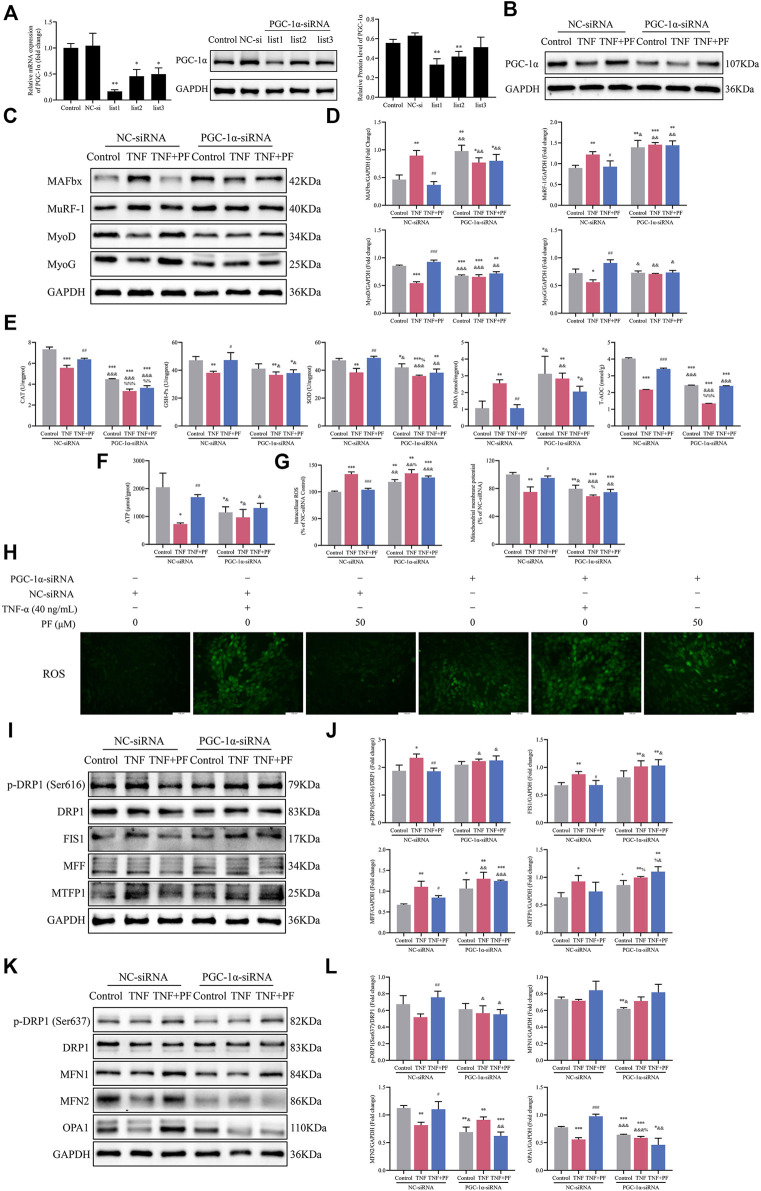
PGC-1α might be important in mediating the effects of PF on TNF-α-treated C2C12 cells. **(A)** The protein and mRNA expression of PGC-1α was detected in C2C12 myoblasts transfected with PGC-1α-targeted siRNA (PGC-1α-siRNA) and nonspecific control siRNA (NC-siRNA). **(B)** Representative immunoblots of PGC-1α expression in NC-siRNA- and PGC-1α-siRNA-transfected C2C12 cells treated with TNF (TNF-α 40 ng/ml) and TNF + PF (PF 50 μM). **(C)** Representative western blots using antibodies against MAFbx, MuRF-1, MyoD, MyoG and GAPDH. **(D)** Western blot analysis. **(E)** The activities of CAT, GSH-Px, SOD, MDA, and T-AOC in C2C12 cells in different groups. **(F)** The expression of ATP in different groups. **(G)** The expression of intracellular ROS generation and Δψm in different groups. **(H)** Intracellular ROS were observed by the DCFH-DA (green) method using an inverted fluorescence microscope in different groups (200× scale bars: 100 μm). **(I)** Representative western blots using antibodies against p-DRP1 (Ser616), DRP1, FIS1, MFF, MTFP1 and GAPDH. **(J)** Western blot quantification. **(K)** Representative western blots using antibodies against p-DRP1 (Ser637), DRP1, MFN1, MFN2, OPA1, and GAPDH. **(L)** Quantification of protein expression. All protein expression was normalized to that of GAPDH as a loading control. The data are presented as the means ± S.D. *n* = 3, ^*^ *p* < 0.05, ^**^ *p* < 0.01, ^***^ *p* < 0.001 compared with the control group or the Control NC-siRNA group. ^#^ *p* < 0.05, ^##^ *p* < 0.01, ^###^ *p* < 0.001 compared with the TNF + NC-siRNA group; ^&^ *p* < 0.05, ^&&^ *p* < 0.01, ^&&&^ *p* < 0.001 compared with the TNF + PF + NC-siRNA group; ^%^ *p* < 0.05, ^%%^ *p* < 0.01, ^%%%^ *p* < 0.001 compared with the Control PGC-1α-siRNA group.

The expression of PGC-1α was decreased after transfection with PGC-1α-siRNA, as confirmed by RT–qPCR and WB data (Figure 8A). At the same time, the effect of PF on enhancing PGC-1α expression was also blocked (Figure 8B). PGC-1α-siRNA transfection obviously increased the expression of MAFbx and MuRF-1 and reduced the expression of MyoD and MyoG. However, knockdown of PGC-1α attenuated the protective effect of PF (Figures 8C,D). Similarly, the effect of PF on improving the oxidative stress induced by TNF-α was largely abrogated by PGC-1α-siRNA (Figure 8E). Moreover, the beneficial effect of PF on ATP production was abolished when PGC-1α was inhibited in C2C12 cells (Figure 8F). Notably, the effects of PF on ROS generation and Δψm were also markedly abolished by PGC-1α downregulation (Figures 8G,H).

We next examined mitochondrial fission proteins and fusion proteins in myoblasts. TNF-α induced an increase in the expression of fission proteins [p-DRP1 (Ser616), FIS1, MFF and MTFP1] and a decrease in the expression of fusion proteins (MFN2 and OPA1) in the NC-siRNA group. However, PF obviously ameliorated the disorder of mitochondrial fission and fusion. As we expected, the beneficial effect of PF was compromised after downregulation of PGC-1α (Figures 8I–L).

## Discussion

In recent years, herbal medicines have attracted a large amount of attention for their potential treatment of CKD. Despite the discovery and extensive application of drug prediction tools for drug effectiveness, there is still a lack of drugs that effectively improve skeletal muscle atrophy in CKD (Huang et al., 2019). Earlier, extensive researcher conducted active ingredients in herbal medicines to control CKD (Chen et al., 2021). PF also provides protection against acute renal injury, diabetic kidney disease, nephrotic syndrome and renal fibrosis (Liu et al., 2016; Wang et al., 2016; Chen et al., 2017; Lu et al., 2017; Jiao et al., 2021). However, the effects and mechanism of PF on CKD skeletal muscle atrophy remain largely unclear.

This is the first study to demonstrate that PF has therapeutic potential on CKD skeletal muscle atrophy through the AMPK/SIRT1/PGC-1α-mediated ubiquitin–proteasome system (UPS), inflammation, oxidative stress and mitochondrial dysfunction. Moreover, we found that PGC-1α was significantly downregulated in the CKD rat model and modulated by SIRT1 and AMPK in TNF-α-treated C2C12 cells. Notably, our results showed for the first time that knockout of PGC-1α reversed the protective effects of PF on muscle atrophy, oxidative stress and mitochondrial dysfunction with evidence from TNF-α-treated C2C12 cells. These data corroborate our initial hypothesis.

There are still many unanswered questions about skeletal muscle atrophy in CKD. Although low muscle mass, low muscle strength and low physical performance are closely related, there is not yet an agreement on which operational criteria to apply when diagnosing muscle atrophy in CKD patients (Sabatino et al., 2021). Muscle mass and physical performance were determined as direct indicators of muscle atrophy in CKD model rats based on our previous studies (Wang et al., 2019; Hu et al., 2020; Liu et al., 2021). The PF-treated group was confirmed by an increase in body weight, muscle weight and muscle fiber CSA. Moreover, we also found that the body weight of the rats in the Model group reached a peak and then began to decrease at the 10th week, with the body weight of the PFL groups increasing slowly throughout the whole process. Currently, the treatments have focused mostly on promoting exercise and improving nutritional intake. The pharmacological options for skeletal myopathy in CKD are still at an early stage of development. In recent years, we have focused on discovering new drugs to treat skeletal muscle atrophy in CKD, including calycosin, atractylenolide III, formononetin and curcumin (Wang et al., 2019; Hu et al., 2020; Wang et al., 2020; Liu et al., 2021). The 5/6 nephrectomy model has been widely used to clarify the pathogenesis of skeletal myopathy in CKD and to explore potential therapeutic targets such as inflammation, oxidative stress and mitochondrial impairment. Our results found for the first time that PF can not only improve kidney function in CKD rats but also regulate calcium and phosphorus disorders. Hyperphosphatemia can trigger and aggravate CKD by stimulating mitochondrial oxidative stress and inducing endothelial inflammation. Hypocalcemia causes excessive secretion of phosphorus and parathyroid hormone, leading to chronic renal failure (CRF) and end-stage kidney disease (ESRD) (Felsenfeld et al., 2015). Kidney disease itself is one of the etiologic factors leading to muscle atrophy in CKD, and PF improved muscle atrophy by improving renal function in CKD rats.

Most intracellular proteins are degraded by the same ATP-dependent system—the UPS. The balance between protein synthesis and degradation is critical for muscle mass and functions (Schiaffino et al., 2013). MAFbx and MuRF-1 are the main muscle-specific E3 ubiquitin ligases that play important roles in the UPS (Bodine et al., 2001; Cao et al., 2018). Under muscle atrophy conditions, the expression of MAFbx and MuRF-1 is largely increased. Mature muscles are composed of muscle fibers, which are covered with myofibrils and satellite cells (Grefte et al., 2007). When muscle fibers are damaged, muscle satellite cells are activated to repair injured muscle fibers or form new. MyoD and MyoG are myogenic markers whose expression decreases when muscle fibers are damaged (Zhang et al., 2010). Our results indicate that CKD significantly increases the expression of muscle protein degradation markers (MAFbx and MuRF-1) and decreases the expression of MyoD and MyoG. PF improved this impaired function. The study offers the same important insights into the effects of PF on the UPS *in vitro*.

In addition, inflammatory cytokines activate myostatin, which induces proteolytic phenotype muscle cells and inhibits satellite muscle cell proliferation, leading to skeletal muscle atrophy in CKD (Bataille et al., 2021). Recent studies (Cao et al., 2011; Kurt et al., 2019) demonstrated that PF reduced the production of proinflammatory cytokines (IL-6, IL-1β, and TNF-α) and increased the anti-inflammatory factor IL-10, which is consistent with the present results in rat serum. Under skeletal muscle atrophy conditions, the imbalance between ROS generation and elimination impairs the redox system, leading to an increase in harmful oxidized products such as MDA and a decrease in the content of T-AOC, CAT, GSH-Px and SOD. These molecules can in turn lead to ROS generation, resulting in muscle atrophy by enhancing the degradation of muscle proteins (Gyurászová et al., 2020; Sabatino et al., 2021). In particular, in this experiment, we found that the activities of MDA and SOD were different for higher and lower concentrations of PF. The measurement of MDA often cooperates with the measurement of SOD. The level of SOD activity indirectly reflects the body’s ability to scavenge oxygen free radicals, while MDA can serve as an indicator of the severity of the body’s cells being attacked by free radicals. Additionally, a recent study demonstrated impaired leg muscle mitochondrial oxidative capacity in patients with CKD (Kestenbaum et al., 2020). Albumin plays an antioxidant role in blood vessels (Taverna et al., 2013). (Hirabayashi et al., 2021) reported that chronic malnutrition with hypoalbuminemia results in increased oxidative damage in skeletal muscle, and our results also confirm this. The present research explores, for the first time, the antioxidant effect of PF on skeletal muscle and TNF-α-treated C2C12 cells.

ETC produces ATP using oxygen through the process of oxidative phosphorylation. Based on skeletal muscle energy requirements, we observed a decrease in skeletal muscle mitochondrial ETC enzyme activities and ATP levels. SDH activity was markedly reduced in the muscles of CKD model rats, which represents the mitochondrial amount and the degree of the tricarboxylic acid cycle. Furthermore, CKD muscles and C2C12 cells display decreased Δψm and reduced mitochondrial bioenergetics. These findings make it important to the field of PF to prevent oxidative damage and the mitochondrial response at a safe dosage in muscle atrophy.

Changes in muscle mitochondrial abundance, structure, and dynamics highlight mitochondrial dysfunction as an underlying mechanism in skeletal muscle wasting in CKD. Although mitochondria are involved in the energy metabolism of all organs, especially skeletal muscle, several questions remain unanswered at present, such as the structure and abundance of skeletal muscle mitochondria (Yazdi et al., 2013). Previous studies have indicated that deleterious alterations in mitochondrial morphology in the CKD group were observed by TEM. Swollen and enlarged mitochondria are considered a symbol of dysfunction and inability to perform normal fusion activities (Navratil et al., 2008). The accumulation of enlarged and highly disordered arrangement mitochondria in skeletal muscle has been demonstrated in aged (Liang et al., 2021), CKD (Wang et al., 2019), cancer (Mao et al., 2021) and obesity (Guo et al., 2021)-induced muscle atrophy. Nevertheless, conflicting experimental results have been observed on lots of occasions. Several studies have provided conflicting results and revealed abnormalities consistent with fewer and smaller mitochondria in CKD-(Wang et al., 2020) and diabetes mellitus-(Wang et al., 2018)-induced muscle atrophy. Therefore, whether mitochondria dilate or contract in muscle atrophy is inconclusive. Our results revealed that the organization of muscle fibers was damaged and that some mitochondria were swollen and characterized by an absence of cristae. Different mitochondrial morphologies are associated with multiple physiological and pathophysiological conditions.

It has been shown that structural changes involving mitochondrial dynamics may result in mitochondrial dysfunction, thereby leading to CKD (Chalupsky et al., 2021). Hence, the balance of mitochondrial dynamics is essential for skeletal muscle and myoblasts. Mitochondrial fission separates damaged or dysfunctional components from networks and ensures natural functioning by transferring dysfunctional mitochondria to mitophagy further (Srinivasan et al., 2017; Tilokani et al., 2018). Our previous studies were partly consistent with these findings regarding mitophagy (Wang et al., 2019; Hu et al., 2020). In this study, we found that phosphorylated DRP1 was activated at serine 616. Since phosphorylation of different residues of DRP1 causes opposing effects, p-DRP1 (Ser616) is the major regulator stimulating fission, while p-DRP1 (Ser637) stimulates fusion, which may suggest enhanced mitochondrial fragmentation in atrophied skeletal muscles of CKD model rats. Notably, MTFP1 expression was in contrast to the results for FIS1 and MFF *in vivo* (Morita et al., 2017). Noticed that the suppression of mTORC1 activity reduces the translation of MTFP1, leading to the altered phosphorylation and localization of DRP. However, how MTFP1 controls DRP1 phosphorylation is an open question. Our previous study provided evidence that the expression of mTOR was downregulated in the CKD model (Wang et al., 2019). In addition, the uremic toxin Hippurate has been shown to damage mitochondria and alter mitochondrial function through the activation of Drp1-mediated mitochondrial fission (Huang et al., 2018). Another study showed that lower HB levels may affect mitochondrial function in patients with CKD (Gamboa et al., 2020), and our results confirm this in the serum of CKD model rats.

Damaged material from impaired mitochondria can be repaired by fusion, loss of mitochondrial fusion has detrimental effects on skeletal muscle, as shown by the reduction in MFN2 and OPA1. In contrast, we have reported that MFN2 and OPA1 are increased in diabetic-induced muscle atrophy (Wang et al., 2018). Nevertheless, the explanation for these different results is not straightforward, as the components of mitochondrial dynamics are closely related, with changes in one system affecting the activation/inhibition of another repair mechanism. Hence, the balance obtained from the inhibition and activation of fusion and fission together is more beneficial and should be considered in future studies.

Here, one noteworthy aspect is the *in vitro* experiment. Initially, in our supposition, the fission and fusion machinery would be activated in cell models similar to rat models, and PF could antagonize fission and promote fusion. The protein expression of p-DRP1 (Ser616), MFF, FIS1 and MTFP1 matched the major conclusions of the *in vivo* experiments. However, from the levels of p-DRP1 (Ser637) and MFN1, it could be inferred that the suppressed expression of MFN2 and OPA1 was greater than that of p-DRP1 (Ser637) and MFN1 in C2C12 myoblasts. The balance between fusion and fission is delicate and intricate. Although DRP1 is an important regulator of mitochondrial dynamics, other factors, such as MFF, FIS1, MFN2, and OPA1, also participate in the process. Synthesizing the above analysis, the reasonable explanation is that the activation of mitochondrial fission might be sharper than fusion. These data indicated that the balance between fusion and fission is shifted toward fission in myoblasts and CKD model rats. PF exerts an effective therapeutic effect on suppressing mitochondrial fission and promoting mitochondrial fusion.

PGC-1α is widely considered to be a critical transcriptional regulator of mitochondrial biogenesis and function. PGC-1α modulates mitochondrial dynamics and bioenergetics and increases mitochondrial mass and function, thereby enhancing the energy supply. The threonine 172 site and its phosphorylation play an essential role in the regulation of AMPK activity (Hirabayashi et al., 2021). The AMPK signaling pathway mediates several beneficial functions, including ATP production, and maintains redox potential, which ensures optimal mitochondrial functioning. SIRT1 has been reported to belong to the sirtuin family, resist metabolic disorders, cancer, and cardiac stress (Cantó and Auwerx, 2012) and block the muscle-specific UPS proteins MAFbx and MuRF-1 (Lee and Goldberg, 2013). Activation of SIRT1 might be beneficial for sarcopenic obesity (Guo et al., 2021; You et al., 2021), malnutrition (Hirabayashi et al., 2021) and glucocorticoid-induced atrophy. Our analysis revealed that the AMPK signaling pathway was activated by PF treatment, further revealing that SIRT1 and PGC-1α, located downstream of AMPK signaling pathway, were also upregulated in CKD muscles. Moreover, PF administration reversed the inhibitory effect of TNF-α and upregulated the proteins in the AMPK signaling pathway. Notably, the specific involvement of the AMPK signaling pathway in C2C12 cells and the effect of PF have been confirmed using inhibitors of AMPK and SIRT1. These protective effects of PF were abolished by PGC-1α-specific downregulation.

## Conclusion

In this study, we demonstrated that paeoniflorin mitigated CKD-related skeletal muscle atrophy and suppressed TNF-α-induced C2C12 myoblast damage. Paeoniflorin played a protective role by suppressing oxidative stress and mitochondrial dysfunction partially through the AMPK/SIRT1/PGC-1α pathway. Regulation of AMPK/SIRT1/PGC-1α-mediated oxidative stress and mitochondrial dynamics by paeoniflorin might be a potential pharmacological approach to target CKD skeletal muscle atrophy.

## Data Availability

The raw data supporting the conclusion of this article will be made available by the authors, without undue reservation.
